# Comparison of two rating scales with the orofacial esthetic scale and practical recommendations for its application

**DOI:** 10.1186/s12955-022-02006-9

**Published:** 2022-09-06

**Authors:** Swaha Pattanaik, Mike T. John, Seungwon Chung, San Keller

**Affiliations:** 1 Department of Diagnostic and Biological Sciences, School of Dentistry, University of Minnesota, 515 Delaware Street Southeast, Minneapolis, MN 55455-0348 USA; 2 Department of Educational Psychology, College of Human Development, University of Minnesota, Minneapolis, MN USA; 3 American Institutes for Research, Chapel Hill, NC USA

**Keywords:** Orofacial esthetic scale, Scaling formats, 5-point numerical rating scale, 11-point numerical rating scale, Oral health, Item response theory, Psychometric properties, Dental patient-reported outcome measure, Patient-centred care, Standardization, Reliability, Validity, Oral health impact profile

## Abstract

**Purpose:**

We compared measurement properties of 5-point and 11-point response formats for the orofacial esthetic scale (OES) items to determine whether collapsing the format would degrade OES score precision.

**Methods:**

Data were collected from a consecutive sample of adult dental patients from HealthPartners dental clinics in Minnesota (N = 2,078). We fitted an Item Response Theory (IRT) model to the 11-point response format and the six derived 5-point response formats. We compared all response formats using test (or scale) information, correlation between the IRT scores, Cronbach’s alpha estimates for each scaling format, correlations based on the observed scores for the seven OES items and the eighth global item, and the relationship of observed and IRT scores to an external criterion using orofacial appearance (OA) indicators from the Oral Health Impact Profile (OHIP).

**Results:**

The correlations among scores based on the different response formats were uniformly high for observed (0.97–0.99) and IRT scores (0.96–0.99); as were correlations of both observed and IRT scores and the OHIP measure of OA (0.66–0.68). Cronbach’s alpha based on any of the 5-point formats (α = 0.95) was nearly the same as that based on the 11-point format (α = 0.96). The weighted total information area for five of six derived 5-point response formats was 98% of that for the 11-point response format.

**Conclusions:**

Our results support the use of scores based on a 5-point response format for the OES items. The measurement properties of scores based on a 5-point response format are comparable to those of scores based on the 11-point response format.

## Introduction

A major reason for dental patients to seek treatment is to enhance their orofacial appearance (OA) [1], which influences their self-esteem and social interactions as OA plays an important role in determining perceived personal beauty and success [2–5]. Research shows, faces with crowding and spacing of teeth appeared less intelligent, beautiful, and sexually attractive, and even socioeconomically disadvantaged to others than the same faces with ideal teeth arrangement [5]. OA or esthetics is thus an important dental patient-reported outcome (dPRO); and one of the four dimensions, or elemental building blocks of the dental patients’ oral health related quality of life (OHRQoL) [6, 7]. Patient-reported OA data would help dental patients and providers in shared treatment decisions[8], consequently, improving dental treatments effectiveness [6, 9] and value-based oral health care [10]. The orofacial esthetic scale (OES) and the Oral Health Impact Profile (OHIP) are the dental patient-reported outcome measures (dPROM) or instruments commonly used to measure OA [6, 9].

The OES was developed in a Swedish prosthodontic patient population [3]. Initially, it was measured on an 11-point numeric rating scale (0 = very dissatisfied, 10 = very satisfied) [3]. Since then, the OES has been translated and adapted for different countries [4, 11–17]. While some of these versions have used the original 11-point response format [4, 14], others have used a more concise 5-point response format (1 = unsatisfactory, 5 = excellent) [11–13]. The 5-point adjectival rating scale is the most widely used response format for dPROMs; in line with medical Patient-Reported Outcome Measures, or PROMs [6].

Application of PROMs with a 5-point response format has conceptual and technical advantages. Compared to an 11-point response format, a 5-point response format is more comprehensible and easier to use, [11] and its conciseness can improve response rate and quality [18]. A technical advantage of the 5-point response format is presence of fewer parameters when the response format data are modeled. However, no studies have compared the properties of data provided by 5-point and 11-point response formats using modern measurement theory.

Currently there is no consensus on the ideal response format for the OES and other dPROMs assessing OA such as the Dental Impacts on Daily Living (DIDL)[19] questionnaire and the Psychological Impact of Dental Aesthetics Questionnaire (PIDAQ) [20]. Hence, efforts toward standardization of OA assessment are hindered. With regard to PROMs in general, a recent review of the evidence concluded that the issue required further empirical study within the context of particular therapeutic areas as results might vary according to disease and therapeutic specialty [21].

The purpose of our study was to compare measurement properties of the 5-point and 11-point response formats for the OES, to determine whether collapsing the 11-point response format to a 5-point response format would degrade OES score precision.

## Methods

### Study population, recruitment, and data collection

We recruited adult dental patients from HealthPartners dental clinics in Minnesota (N = 2,115). Removing individuals who did not respond to the OES items leaves N = 2,078. Details about data collection and recruitment have also been provided in previous research papers [4, 16]. Our sample size satisfied sample size recommendations (of 500 or greater) for the Item Response Theory (IRT) model that we used in our analysis [22].

### Measure: orofacial esthetic scale

Details of the OES development have been published elsewhere [3] and are briefly summarized here. The OES consists of seven items addressing specific esthetic components (face, facial profile, mouth, rows of teeth, tooth shape/form, tooth color, gums) and one item assessing the overall impression (Table 1). Originally, the response format was a 0 to 10 numeric rating scale, anchored only with “very dissatisfied” and “very satisfied” (with appearance) at the extremes of 0 and 10, respectively. Scores of items 1 through 7 can be summed up to form an OES summary score that can range from 0 through 70, with higher scores representing less impaired esthetics [3, 16]. The eighth item represents an overall impression of OA and no specific esthetic component, so it is not included in any of the subscale scores. The OES was initially tested among Swedish prosthodontic patients [3]. Since then, the validity of OES scores has been assessed for other dental patients [4, 16], and general populations [23] in several other countries.

**Table 1:** OES and OHIP items

OES
How do you feel about the appearance of your face, your mouth, your teeth and your replacements (prostheses, crowns, bridges and implants)?
*0: Very dissatisfied-10: Very satisfied*
1. Your facial appearance
2. Appearance of your facial profile
3. Your mouth's appearance (smile, lips, and visible teeth)
4. Appearance of your rows of teeth
5. Shape/form of your teeth
6. Color of your teeth
7. Your gum's appearance
8. Overall, how do you feel about your face, your mouth and your teeth?
**OHIP**
*0:Never-10: Very Often*
3. Have you noticed a tooth which doesn't look right?
4. Have you felt that your appearance has been affected because of problems with your teeth, mouth, or dentures?
19. Have you been worried by dental problems?
20. Have you been self-conscious because of your teeth, mouth, or dentures?
22. Have you felt uncomfortable about the appearance of your teeth, mouth, or dentures?
31. Have you avoided smiling because of problems with your teeth, mouth, or dentures?

^*^OHIP items are numbered in the same way as in the original questionnaire

### Additional measure: oral health impact profile

Details of OHIP development have been published elsewhere [24] and are briefly summarized here. The OHIP is the most widely used instrument to measure OHRQoL in adults with oral conditions [6]. It is a more comprehensive instrument than the OES. While the OES only measures OA, the OHIP measures seven conceptual dimensions of impact corresponding to Locker’s model of Oral Health [25], which is based on the World Health Organization’s (WHO’s) s International Classification of Impairments, Disabilities, and Handicaps from 1980 [26]. The dimensions of impact are functional limitation, physical pain, psychological discomfort, physical disability, psychological disability, social disability, and handicap. Originally, the OHIP questionnaire had 49 items [24] organized into the seven dimensions. Later, researchers developed 14- and 5-item versions [27, 28]. Based on previous exploratory [29] and confirmatory [30] factor analysis results from previous studies, there are six items (3, 14, 19, 20, 22, 31) that capture OA as an underlying factor or dimension in the 49-item OHIP (see Table 1). We used the six-item indicators of the OA OHIP scale in our analysis.

For each question, respondents are asked to indicate on a 5-point Likert scale (0- never, 1- hardly ever, 2-occassionally, 3-fairly often, and 4-very often) according to how frequently they experienced each problem within the past twelve months. Respondents may also be offered a "don't know" option for each question. All impacts in the OHIP are conceptualized as adverse outcomes, thus, a higher score indicates more negative impacts of oral health problems. Overall OHIP scores are computed in two ways. The simpler scoring method is to sum all 49 unweighted items. The second method is to standardize the seven subscale scores and then sum those standard scores.

### Statistical analysis

**Fig. 1:**
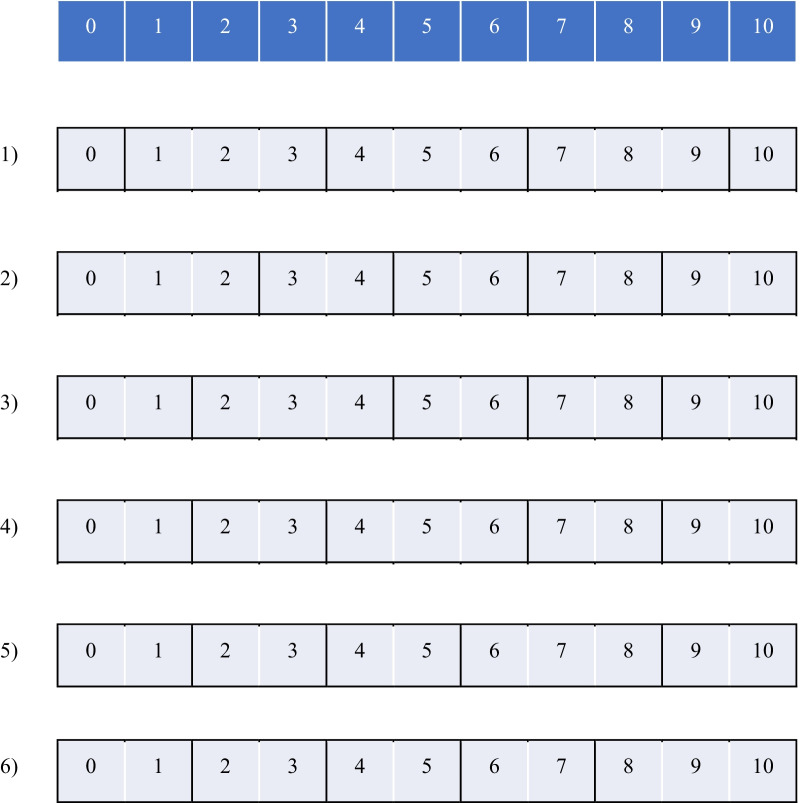
The six derived 5-point response formats

The hypothesis of our study was-when a 11-point response format is collapsed to a 5-point response format, psychometric properties of OES scores will not be compromised. Multiple options for the 5-point response format exist if the study is designed to compare the 11-point response format with a "derived" 5-point response format. Thus, as the first step, we defined several “plausible” 5-point response formats to be investigated in the study, each created by a different method of collapsing the 11-point response format. A challenge was that the 11 points be assigned relatively evenly among five categories. Hence, we set up two simple principles for grouping categories within the 11-point response format: *Rule 1* was to disallow 4-category grouping, and *Rule 2* was to disallow 1-category grouping with *Exception* (1-category is allowed) at the beginning and the end of the response format. *Rules 1* and *2* yielded balanced response groups, meaning that only groupings of 2- and 3-categories existed. Note that *Exception* corresponds to Patient-Reported Outcomes Measurement Information System (PROMIS) guidelines [31]. Following *Rules 1* and *2* coupled with *Exception*, we obtained the six “derived” 5-point response formats (see Fig. 1). Response options of the 11-point response format were collapsed into fewer response options in a manner that any imbalance could be avoided. Our approach was in line with how response options are grouped together for the pain rating scales [32].

### Descriptive analysis

For the 11-point response format, we plotted histograms for Item 1–8 to examine the frequencies in each response option.

### Classical test theory (CTT)

#### Reliability analysis (Internal consistency)

We computed Cronbach’s alpha [33] for the 11-point response format and six derived 5-point response formats to assess any changes in OES reliability. Also, we used a Bootstrap confidence interval for Cronbach’s alpha because the distribution of item scores could not be well approximated by a normal distribution.

#### Validity analysis (Correlation analysis based on sum scores)

We computed Spearman’s rank correlation coefficients between the 11-point response format and the six derived 5-point response formats based on the observed scores (raw scores) for the seven items addressing specific esthetic components as well as the summary score. If the correlation is high (r > 0.95), then we can infer that there is a close similarity in the scores between the two response formats. Also, within each response format, we computed the correlation between the aggregated seven items and a global item assessing overall impression. If these correlations were similar in size, indicating a similar relationship to overall OA, we could assume the scores based on the different response formats have a similar interpretation and so are measuring the same “construct.” Furthermore, we computed correlations between summed scores of the 11-point and 5-point response formats of the OES, and that of the OA indicators from the OHIP to determine whether the relationship of the scores to the external criterion was invariant across the two response formats.

### Confirmatory factor analysis (CFA)

#### Reliability analysis (Internal consistency)

We also derived the composite reliability estimate or Mcdonald's omega coefficient [34], which is an “indicator of the shared variance among the observed variables used as an indicator of a latent construct” [35].

### Item response theory (IRT)

Item Response Theory (IRT) is a psychometric theory that refers to a family of associated statistical models that predict responses to a given set of items based on each item’s properties and the respondent’s position on continuum of latent trait of interest (OA) measured by the scale (OES) [36]. Unidimensionality of the scale is required in order to perform IRT based analysis. Previous studies have supported unidimensionality of the OES [3, 16]. Samejima’s graded response model (GRM) was used for calibration of our items [37]. This model is suitable for ordered scoring categories, which is the case for the OES. GRM specifies the probability of responding to a particular category or higher, versus responding to lower categories for each value of latent variable (trait)$\theta$, which is (perceived) OA in our study. In GRM, each item is characterized by one slope parameter, and category threshold or location parameters at which the probability of responding to a particular category or higher is 0.5. Note that the number of category threshold parameters for an item equals one less than the number of categories. GRM is considered an extension of the two-parameter logistic (2PL) model for binary data, which is characterized by two parameters, i.e., slope and location parameters. While other models can be used for polytomous items with ordinal data, GRM is a popular model in research with health-related outcomes [38]. Also, we thought GRM is more appropriate than other models extending one-parameter logistic (1PL) model to ordinal data, which assumes equal slope parameters across items, because the slope parameter estimates were varying across items for the OES. With the GRM parameters, we can derive category response curves (CRCs). A CRC represents the probability of responding in a particular category as a function of trait level$\theta$. We fitted a GRM to the 11-point response format (0 = very dissatisfied, 10 = very satisfied) and the six derived 5-point response formats.

#### Reliability analysis (Item/Test information)

Information is analogous to reliability of measurement, and it is provided both at item and test (scale) level. An item information function or curve shows the amount of (Fisher) information an item contains along the continuum of a latent trait, i.e., OA [39]. CRCs from GRM can be transformed into an item information function. Multiple factors contribute to item information for polytomous models. For GRM, magnitude of the slope parameter, and the distance between the category thresholds or location parameters determine the amount of information. The test (or scale) information curve is obtained by simply summing the item information curves. Also note that the information function is related to measurement precision. Specifically, (conditional) information is inversely related to standard error of measurement (SEM) [40].

Furthermore, we computed the total information area (TIA), which represents the area under the test (or scale) information. To account for differential contribution due to unequal number of respondents along the latent trait continuum, we weighted the TIA with the proportion of respondents in each interval of the latent trait. Specifically, we divided the latent trait ranging from − 4 to 4 into 8 intervals with equal length and then obtained the proportion of the total respondents within each interval. This served as a “weight” to be multiplied by the average information within the corresponding interval. We will term this index “weighted total information area (TIA).”

#### Validity analysis (Correlation analysis based on IRT scale scores)

We estimated the IRT scores using the GRM for each response format. The IRT scores refer to person location estimates from an IRT model. In IRT scoring, a respondent’s location on the OA continuum is obtained by utilizing the respondent’s item response pattern coupled with estimated item parameters [39]. Specifically, we obtained the *expected *a posteriori (EAP) scores [41]. EAP uses the mean of the posterior distribution as the latent traits. Then, we calculated the correlation between the IRT scores based on the 11-point response format and each of the six derived 5-point response formats. Furthermore, we computed correlations between the EAP scores from the 11-point response format and the derived 5-point response formats of the OES and those from the OA indicators of the OHIP. Note that the analysis is identical to what we described above for the CTT framework, but now the correlation analysis was performed using the scores from IRT analysis instead of sum scores. All analyses were performed using the *mirt* package in R [42].

## Results

### Descriptive analysis

**Fig. 2:**
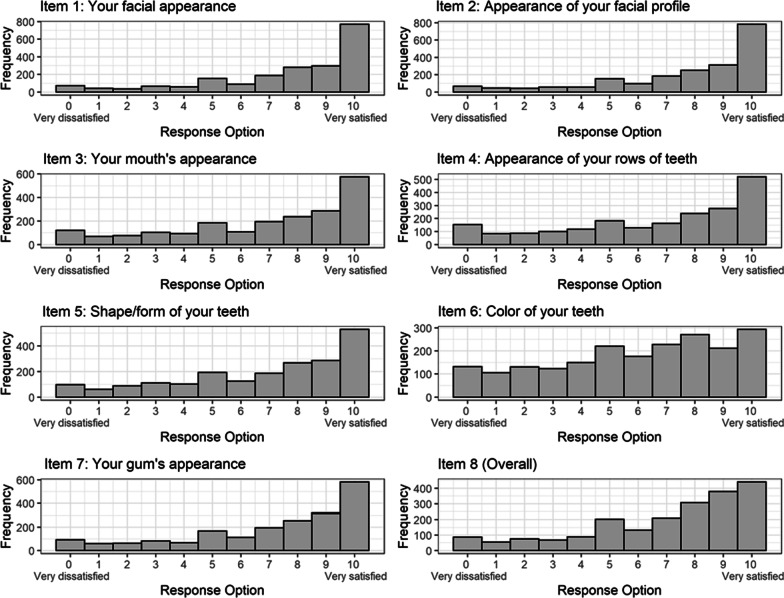
Histograms of Items 1–8 on an 11-point response format

Our sample consisted a total of 2,078 study participants. There were more females (n = 1,240) than males (n = 838) participating in the studies. The mean age of the participants was 54.68 ± 16.18 (range 22–97) years. Table 2 shows descriptive statistics including the five number summary for each OES item. The mean values ranged from 5.92 to 7.72. Figure 2 shows histograms of the 11-point response format for Items 1–8. Generally, they show a left-skewed distribution. Category 10 shows the highest frequency, suggesting that a majority of respondents were “very satisfied” with each component of OA (Items 1- 7), and were “very satisfied” overall with their OA (Item 8). Interestingly, patients’ responses to Item 6 (“Color of your teeth”) was relatively evenly spread.

**Table 2:** Descriptive statistics with the OES items

Variable	Mean	Standard Deviation	Minimum	Lower quartile	Median	Upper quartile	Maximum
oes1 (facial appearance)	7.69	2.76	0	6	9	10	10
oes2 (facial profile)	7.72	2.76	0	6	9	10	10
oes3 (mouth’s appearance)	6.86	3.14	0	5	8	10	10
oes4 (appearance of rows of teeth)	6.55	3.26	0	4	8	10	10
oes5 (shape and form of teeth)	6.84	3.04	0	5	8	10	10
oes6 (color of teeth)	5.92	3.08	0	4	6	8	10
oes7 (gingiva’s appearance)	7.17	2.98	0	5	8	10	10
oes8 (global item)	7.00	2.86	0	5	8	9	10

### CTT

#### Reliability analysis (Internal consistency analysis)

Cronbach alpha estimates for the 11-point response format and six derived 5-point response formats with their 95% confidence intervals (CIs) are presented in Table 3. We observe that the alpha estimates of the 5-point response formats barely decreased. The alpha estimate from the 11-point response format was 0.95, and the estimates from the 5-point response format were 0.94 in all six possible response formats.

**Table 3:** Cronbach alpha estimates for the 11-point response format and the six derived 5-point response formats

Response format	Alpha
11-point	0.95 (0.94, 0.95)
5-point (Option 1)	0.94 (0.94, 0.95)
5-point (Option 2)	0.94 (0.93, 0.95)
5-point (Option 3)	0.94 (0.94, 0.95)
5-point (Option 4)	0.94 (0.94, 0.95)
5-point (Option 5)	0.94 (0.93, 0.95)
5-point (Option 6)	0.94 (0.93, 0.94)

#### Validity analysis (Correlation analysis)

Spearman’s rank correlation coefficients between the 11-point response format and the six derived 5-point response formats based on the raw scores are presented in Table 4. The first seven columns show the item correlation between the response formats for Items 1–7, and the last column is the correlation based on the summary score of the 7 items. The summary score correlation was well above 0.97 across all the 5-point response formats (Options 1–6), suggesting that there is a very strong relationship between the two response formats. The correlation examined by each item also indicates that the 5-point response formats are highly correlated with the 11-point response format.

**Table 4:** Spearman’s rank correlation coefficient between the 11-point response format and the six derived 5-point response formats based on item scores and summary scores

	Item 1	Item 2	Item 3	Item 4	Item 5	Item 6	Item 7	Summary Scores
Option 1	0.97	0.98	0.98	0.98	0.97	0.97	0.97	0.99
Option 2	0.95	0.94	0.97	0.97	0.97	0.98	0.96	0.99
Option 3	0.95	0.94	0.97	0.97	0.97	0.98	0.96	0.99
Option 4	0.95	0.94	0.97	0.97	0.97	0.98	0.96	0.99
Option 5	0.94	0.94	0.96	0.97	0.97	0.98	0.96	0.98
Option 6	0.87	0.87	0.93	0.94	0.93	0.97	0.91	0.97

Pearson correlations mirror the Spearman correlations

In addition, correlations between the aggregated items (Item 1—7) and the global item (Item 8) and their 95% CIs are presented in Table 5. Please note that except for Table 5 all analysis was done focusing on the summary score of the items from 1–7 within the OES. The correlation was 0.92 for the 11-point response format, and it ranged from 0.85 to 0.89 for the six derived 5-point response formats. Overall, the difference was minimal between the 11-point response format and any of the six derived 5-point response formats. Given that the correlations were similar in magnitude, we determine that the relationship between the global item score and the seven-item composite scores remain largely the same even after collapsing 11 response categories to 5. In other words, the six derived 5-point response formats are measuring the same “construct”.

**Table 5:** Spearman’s rank correlation coefficient between the aggregated items (Item 1–7) and the global item (Item 8) and their 95% CIs

	r (95% CI)
11-point	0.92 (0.91–0.92)
5-point (Option1)	0.88 (0.87–0.89)
5-point (Option2)	0.89 (0.88–0.90)
5-point (Option3)	0.89 (0.88–0.90)
5-point (Option4)	0.89 (0.88–0.90)
5-point (Option5)	0.88 (0.87–0.89)
5-point (Option6)	0.85 (0.84–0.86)

Pearson correlations mirror the Spearman correlations

Table 6 shows estimated correlations and their 95% CIs between the summed scores from the 11-point response format and the six derived 5-point response formats of the OES and those of the OA indicators of the OHIP. The sum scores from the two response formats (11-point and the six 5-point response formats) of the OES correlate similarly with the sum score of the OA dimension of the OHIP for the six indicators. We observe negative correlation estimates as the scoring system of the OES is inverse to that of the OHIP. While for the OHIP the higher the score means worse OA (‘bad’ OA), for the OES, higher score means better OA (‘good’ OA).

**Table 6:** Spearman’s rank correlations between the sum scores of the OES scales (11-point and the 5-point response formats) and the external measure (OA from OHIP) and their 95% CI

	OA from OHIP (6 items)
11-point	− 0.68(− 0.71, − 0.66)
5-point (Option1)	− 0.67(− 0.69, − 0.64)
5-point (Option2)	− 0.67(− 0.7, − 0.65)
5-point (Option3)	− 0.67(− 0.70, − 0.65)
5-point (Option4)	− 0.67(− 0.69, − 0.64)
5-point (Option5)	− 0.67(− 0.70, − 0.65)
5-point (Option6)	− 0.68(− 0.70, − 0.65)

Pearson correlations mirror the Spearman correlations

### Confirmatory factor analysis (CFA)

#### Reliability analysis (Internal consistency)

McDonald's omega estimates for the 11-point response format, and six derived 5-point response formats with their 95% confidence intervals (CIs), are presented in Table 7. We found the omega estimates were similar to the alpha estimates; and the omega estimates for the 5-point response formats barely decreased. The omega estimate from the 11-point response format was 0.95, and the estimates from the 5-point response format were 0.94 in all six possible response formats.

**Table 7:** McDonald's omega estimates for the 11-point response format and the six derived 5-point response formats

Response Format	Omega (95% CI)
11-point	0.95 (0.94, 0.95)
5-point (Option 1)	0.94 (0.94, 0.95)
5-point (Option 2)	0.94 (0.94, 0.95)
5-point (Option 3)	0.94 (0.94, 0.95)
5-point (Option 4)	0.94 (0.94, 0.95)
5-point (Option 5)	0.94 (0.94, 0.95)
5-point (Option 6)	0.94 (0.93, 0.94)

### IRT

#### Reliability analysis (item/test information)

**Fig. 3:**
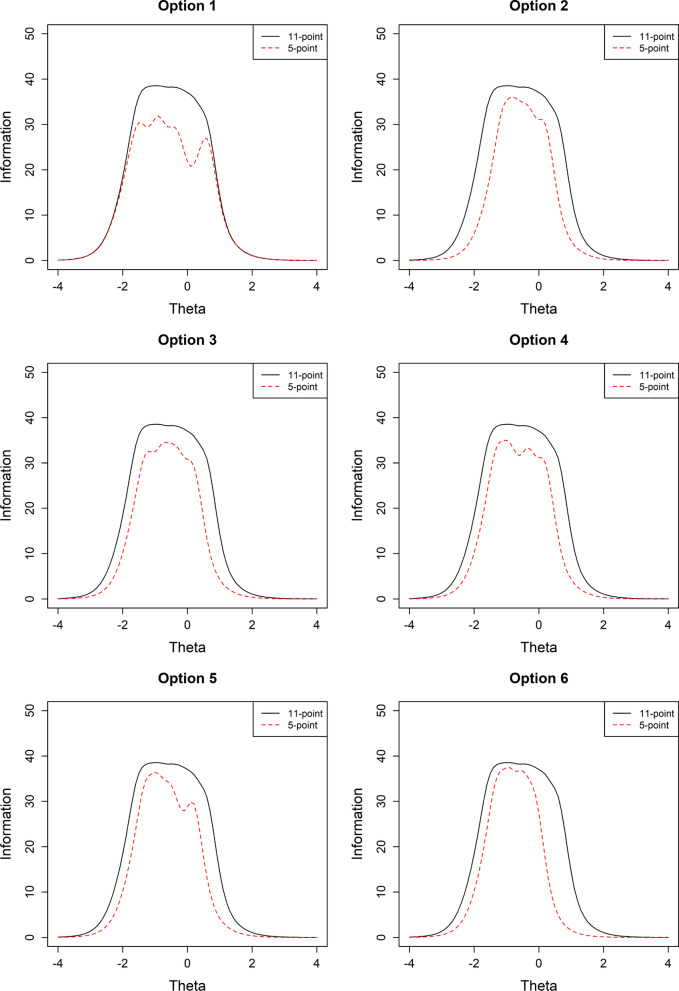
Test information function curves for the six-derived response format options of the 5-point scale

We compared the test (or scale) information functions of the 11-point response format and the six 5-point response formats to examine the loss of information when a 5-point response format is used at the scale level (Fig. 3). We found that some loss of information occurred when going from the 11-point response format to the 5-point response format. The shapes of information functions for the six 5-point response formats differed. Option 1 showed loss of more information in the middle range ($\theta$ level between − 1.5 and 0.5), with the greatest information loss occurring at $\theta$ around 0. The other 5-point response formats (Options 2–6) showed relatively similar patterns in the way the information curves for the 5-point response formats were shrunken compared to that of the 11-point response formats. The loss of information was relatively even across the range of the latent trait ($\theta )$.

Examining the weighted TIA (Table 8), we found that the information of the 5-point response format resulted in above 98% of that of the 11-point response format for all of the collapsed options except Option 6. In Option 1, even though loss of information appeared substantial in the middle range of latent trait ($\theta$) (see Fig. 3), the TIA when weighted by the unequal distribution of respondents was nearly the same as that of the 11-point response format. On the other hand, Option 6 where the loss of information occurred for the high latent trait ($\theta$) resulted in a relatively greater reduction in the proportion of the weighted TIA due to the left-skewed distribution, as shown in Fig. 2. However, even for Option 6 where the information loss was the highest, we observed about 88% of the information provided by the 11-point response format.

**Table 8:** Weighted total information area (TIA) for the OES with the 11-point item response format compared to the OES with the six derived 5-point response formats

	TIA	Ratio (5-point/11-point)
11-point	29.52	
5-point (Option1)	29.16	0.99
5-point (Option2)	28.93	0.98
5-point (Option3)	29.15	0.99
5-point (Option4)	29.15	0.99
5-point (Option5)	29.12	0.99
5-point (Option6)	26.07	0.88

### Validity analysis (IRT Scoring)

IRT scores were estimated, and the scores of the six 5-point response formats were compared against those of the 11-point response format. The correlations between the EAP scores of the 11-point response format and those of the 5-point response format and their 95% CI are displayed in Table 9. For Option 1, the correlation is almost 1, and the other options also show high correlations ranging from 0.93 to 0.96. As expected by the weighted TIA, Option 6 showed the lowest correlation. Nevertheless, correlation was greater than 0.90 in all the scenarios.

**Table 9:** Correlations between the EAP scores of the 11-point response format and the six derived 5-point response formats and their 95% CI

	r (95% CI)
Option 1	0.99 (.99, .99)
Option 2	0.96 (.95, .96)
Option 3	0.96 (.96, .96)
Option 4	0.96 (.96, .96)
Option 5	0.96 (.96, .96)
Option 6	0.93 (.92, .93)

Table 10 displays estimated correlations and their 95% CIs between the EAP scores from the 11-point format and the six derived 5-point formats of the OES along with those of the OA indicators of the OHIP. It showed that both response formats have nearly identical correlations with the external measure.

**Table 10:** Correlations between the EAP scores of the OES (11-point response format and the 5-point response format) and the external measure (OA from OHIP) and their 95% CI

	OA from OHIP (6 items)
11-point	− 0.66 (− 0.68, − 0.63)
5-point (Option1)	− 0.66 (− 0.68, − 0.63)
5-point (Option2)	− 0.66 (− 0.68, − 0.63)
5-point (Option3)	− 0.66 (− 0.68, − 0.63)
5-point (Option4)	− 0.66 (− 0.68, − 0.63)
5-point (Option5)	− 0.66 (− 0.68, − 0.63)
5-point (Option6)	− 0.67 (− 0.69, − 0.65)

## Discussion

On rigorous testing of the research hypothesis using CTT- and IRT-based approaches; we found that the measurement properties of the OES were not compromised when an 11-point response format was collapsed to a 5-point response format. The internal consistency analysis showed that scale reliability hardly decreased when the number of response categories was reduced. Also, the correlation analyses based on observed or raw scores showed that scale validity was not undermined. Specifically, we found a strong linear relationship between the summary scores of the 5- and 11-point response formats. The item score correlation results also supported similarity between the two response formats. Additionally, we observed high correlations between the seven OES items and the global assessment item across the 11- and 5-point response formats, implying that both measured the same construct (OA). We also found that both the response formats of the OES correlated well with the external criteria, that is the OA indicators of the OHIP.

We scrutinized item and test (or scale) information for both the response formats to assess IRT-based reliability and found some loss of information for the 5-point response format. This was expected when reducing the number of response options, given that each response category provided information for polytomous items. Considering the relationship between information and SEM; loss of information meant decrease in precision of measurement, and in reliability. Importantly, the IRT analysis helped pinpoint where information loss occurred heavily, as information is given as a function of latent trait and pertinent to individual score [43]. We evaluated six 5-point response formats created by collapsing categories in different manners. While the location and the amount of information loss differed across the six 5-point response formats, the general trend was that scale reliability was sacrificed to a limited extent when using the 5-point response format. However, examining the impact of loss of information on individual scores, we observed that it was overall not meaningful for the IRT-based scores, particularly the EAP scores. For all the 5-point response formats, the correlations between EAP-scores for the 11-point response format with any of the 5-point response formats were greater than 0.9.

In general, the optimum number of response categories in rating scales has been widely debated, yet there is no consensus on the best scaling format [11, 44]. Coarser scales (with fewer response categories) tend to lower the discriminating power that the respondents might be capable of, while finer rating scales (with several response categories) may go beyond their discriminating ability [44]. Previous researchers investigating an optimal response format found that increasing the number of response categories did not necessarily improve scale reliability and validity [44]. The specific number of response categories beyond which increases in scale reliability and discrimination become negligible, has also been a contentious issue [45–47]. Garner explained that this number beyond which there will be no improvement in the scale discrimination, is a function of the amount of discriminability inherent in the items rated [46]. Maydeu-Olivares et al. concluded that the choice of psychometric framework also influences the effect of response format on the reliability and validity of scores [48]. For example, within the IRT framework, they suggested that applied researchers consider factors such as the number of items in an instrument, the items’ discriminating ability, and the goodness of fit of the model in selecting the optimal response format [48].

Previous researchers have successfully applied a 5-point OES to clinical settings [11–13]; in fact, Persic and colleagues strongly recommended its use due to practical benefits for face-to-face and telephone interviews [11]. However, unlike our study, these previous researchers did not perform a comparative analysis of the 11-point to the 5-point response format. Ours is the first study to conduct an in-depth comparison of these two scaling formats commonly used for responses to the OES and other dPROM items. Within the area of patient (medical) reported outcomes, researchers have compared different response formats for a given scale [48–50], using a methodological approach that differed from ours. For example, Hendriks et al. and Garratt et al. concluded that compared to the 10-point response format, the 5-point response format produced better quality data with fewer missing data, more variance, distributions with less skew and kurtosis [49] and lower floor and ceiling effects [50].

### Strengths and limitations

We compared measurement properties of item- and total scores based on responses to the 11-point response format with scores based on responses to six plausible 5-point response formats. The 5-point response formats were derived from collapsing the response categories on the 11-point response format. Our study may be limited due to this research design, as we did not administer both the response formats separately to the patients. Maydeau-Olivares used a repeated measures design [48] where a group of students was divided into two samples. Each sample received a test battery consisting of four instruments, with a target questionnaire that was administered three to four times, each time with a different number of response alternatives. This design helped them capture variability in measurement properties due to respondent in addition to that due to number of response alternatives. Other researchers randomized the patients in their study to receive either a 5-point response format or a 10-point response format [50]. This design helped them compare the quality of data yielded by the two response formats under conditions similar to the way the questionnaire would be administered clinically–that is, in a clinical setting, each patient would receive a single type of response format. By contrast, the limitations of working with “derived” 5-point response formats are that we cannot determine the variability in data quality due to respondent and the impact on the data quality during the actual administration of the collapsed response options [48–51]. On the other hand, an advantage of our study design was that similar to Maydeau-Olivares, we controlled for the “respondent effect”, however, unlike Maydeau-Olivares, we did not need to consider factors such as the influence of test–retest time on the results. Another strength specific to our own study is that we examined six, 5-point response formats instead of choosing just one, which, by providing results from all possible “reasonable” scenarios, increases the generalizability of our results.

We also acknowledge that the study findings are limited by the instrument (or dPROM) we chose to examine. Although our findings evidence the reliability and validity of the 5-point response format, more methodological work is needed to establish its suitability for other dPROMs. Also, we specifically compared the 5- and 11- point response formats because these are commonly used in clinical settings [48, 49]. Additional research will be needed if researchers are interested in fewer than five response alternatives. We could have taken an exploratory approach and determined if some of the categories in the 11-point response format could have been collapsed by examining whether certain CRCs were subsumed by adjacent CRCs. Instead, we adopted a confirmatory approach to specifically address the increasing application of the 5-point response format over the 11-point response format in clinical and research settings [19, 52]. Dental practitioners and researchers already recognize the practical benefits of using the 5-point response format [11, 19]. Our findings further assure them that using a more concise 5-point response format does not compromise the scale reliability, and that the loss of information is limited and not clinically relevant. Since our research purpose was to only compare two response formats that are commonly used with the OES we decided not to go into detail about other properties of the instrument such as redundancy. Previous studies [3, 4, 14–16] can be referred to for additional information on OES development. The robustness of our study findings is supported by the use of a large (N = 2,078) sample of dental patients. A large sample size is required to obtain stable item parameter estimates. We also used IRT and CTT methods, as each have their advantages and disadvantages. In general, previous studies suggest that different psychometric frameworks (e.g. IRT versus CTT) can produce discrepant findings [48]. We believe the different frameworks provided complementary information thus, adding to the strength of our study.

### Significance of the study; recommendations for research and practice

The 5-point response format clearly has several practical and technical advantages over the 11-point response format, making it easier to implement dPROs necessary for pursuing evidence-based dentistry across dental disciplines [53, 54]. Firstly, fully labeled scales are more reliable than partially labeled scales [55]. The current 11-point response format provides label on the first and last category only. Secondly, when researchers employ an IRT framework to evaluate the precision of question responses, they would have fewer parameters to estimate with the 5-point response format compared to the 11-point response format. This would reduce the number of items and responses required to derive stable parameter estimates. Maydeu-Olivares et al. recommended that applied researchers use fewer response alternatives if they are concerned with the goodness of fit of their model and want to be confident that their latent trait estimates are highly reliable [48]. Although, the 11-point response format may help capture patient experiences more comprehensively, it may overestimate precision of patients’ responses. Clinically, a 5-point response format is less burdensome and time-consuming for respondents [18], considering that there are limits to respondents’ capacity to process or discern a large number of response categories [55, 56]. It is also easier for clinicians to administer the 5-point response format, especially when they are reading aloud the response categories to their patients who might need assistance with filling out surveys such as the elderly and those with low literacy level [11, 57]. Such verbal clarification becomes more impractical with increasing number of response categories such as in the 11-point response format [57]. Researchers might be inclined to use more number of response categories to maximize reliability (precision) [48], however, evidence shows that patients are often reluctant to use all the response categories [50] resulting in response biases such as going for extreme or neutral responses.

Currently, there is no consensus on the most appropriate number of response categories for the OES and our study offers promising evidence to support broader application of the 5-point response format. Additional research using a randomized or a repeated measures design will help account for any issues that might occur during the administration phase. We used the OHIP as an external measure to further support our findings. The OES and OHIP are both dPROMs that capture OA. Our study showed that the 11-point and 5-point response formats with the OES correlated well with the OA item indicators within the OHIP, suggesting that both scales measure the same construct. While researchers may use the OES if they need a stand-alone instrument that specifically measures OA; they may use the OHIP for a broader perspective of OA in context of other components of the patients’ overall oral health experience.

We found that the OES score precision is not degraded when its response categories are reduced. Our research findings are thus an important step toward standardizing response formats for the OES and subsequently other dPROMs in the future. We believe the use of standardized dPROMs would enhance communication among dental professionals about the impact of oral diseases on their patients. It would also improve dentist-patient communication and may help patients accept and adhere to the recommended treatment plan more readily [8]. Our analytical procedures offer guidance for conducting similar investigations for other dPROMs. Although further methodological work is needed, our study findings pave the way for standardization efforts with the OES and possibly other dPROMs in the future.

## Conclusion

To conclude, our study findings are highly encouraging for clinicians and researchers in the dental community who would like to use a 5-point response format for responses to the OES items. Our results showed high correlations between OES scores based on the 5-point response format and OES scores based on the 11-point response format, and the latent scores of the majority of the respondents were recovered well with all of the six derived 5-point response formats. From a psychometric point of view, OES scores based on an 11-point response format were equivalent to those based on a 5-point response format, hence, using the 5-point response format instead of the 11-point response format would have a negligible impact on OES score reliability and validity. The evidence we provide along with the practical and technical advantages of using a more concise 5-point response format, alleviates any concerns that the psychometric properties of OES scores would be compromised by collapsing the 11-point response format categories into 5.

## Data Availability

The datasets during and/or analysed during the current study available from the corresponding author on reasonable request.
